# The Epidemics of Donations: Logistic Growth and Power-Laws

**DOI:** 10.1371/journal.pone.0001458

**Published:** 2008-01-23

**Authors:** Frank Schweitzer, Robert Mach

**Affiliations:** Chair of Systems Design, ETH Zurich, Zurich, Switzerland; University of East Piedmont, Italy

## Abstract

This paper demonstrates that collective social dynamics resulting from individual donations can be well described by an epidemic model. It captures the herding behavior in donations as a non-local interaction between individual via a time-dependent mean field representing the mass media. Our study is based on the statistical analysis of a unique dataset obtained before and after the tsunami disaster of 2004. We find a power-law behavior for the distributions of donations with similar exponents for different countries. Even more remarkably, we show that these exponents are the same before and after the tsunami, which accounts for some kind of universal behavior in donations independent of the actual event. We further show that the time-dependent change of both the number and the total amount of donations after the tsunami follows a logistic growth equation. As a new element, a time-dependent scaling factor appears in this equation which accounts for the growing lack of public interest after the disaster. The results of the model are underpinned by the data analysis and thus also allow for a quantification of the media influence.

## Introduction

The tsunami that infested South-Eastern Asia on 26 December 2004 has not just caused a tremendous death toll and destruction, but also a huge outpouring of donations worldwide to support relief for the affected areas. The fact that both the number and total amount of donations summed up in an unprecedented way was of course induced by the dimension of the disaster. It also benefited from social feedback processes, caused by the massive involvement of the mass media which eventually led to social herding in donating money.

Herding behavior plays an important role in biological but also in social systems. It is governing biological swarming [1], [2], as well as investment strategies in financial markets [3], [4] r collective opinion formation [5], [6]. The underlying mechanism of transmission of influence from one individual to another can be found in a large class of so-called contagion models [7], which also cover epidemic models [8], [9], [10], in particular the SIR (susceptible-infected-recovered) model [11], [12]. A prominent example to link herding behavior and epidemic dynamics is found in the spread and adoption of innovations [13] and fashion [14]. While many odels in sociology, economics, and political science [15], [16], [17], [18] assume a threshold for the adoption of new technologies or behavior, the SIR model is called an independent interaction model [7] because contagion occurs with a propability independent of the history of exposures.

In this paper, we apply the concept of contageous behavior to the collective dynamics of donations after the tsunami catastrophe. Thanks to the availability of a unique database described below, we are able to quantify these dynamics. The statistical analysis reveals a power-law behavior for the distributions of donations with similar exponents for different countries and, even more remarkably, both before and after the disaster. We further show that the dynamics of donations follow a logistic growth already known from models of epidemic spreading. As a new element of this dynamics, a time-dependent contagion rate appears which describes the mean-field interaction provided by the mass media. The considerable decrease of this influence in time accounts for the growing lack of public interest after the disaster. By deducing it from the data available, we are able to quantify the influence of the media reporting about the tsunami.

## Materials and Methods

### Analysis of donation time series

Individual donations, as a voluntary act, may depend on individual, cultural, organisational and economic conditions and thus may differ between countries. In order to find out statistical similarities in the distribution of donations, we investigated three different time series from donor organizations in Germany (DH, AH) and Switzerland (GK) summarized in Table 1.

**Table 1:** Summary of data sets obtained from different donor organizations: (DH)–“Deutschland hilft” (Germany), (AH)–“Andheri-Hilfe” (Germany), (GK)–“Glückskette” (Switzerland).

Donor org	DH	AH	GK
Time int	04/07/26–04/12/23	03/12/29–04/06/30	N/A
*A_tot_*	209,928	1,587,442	N/A
*N_tot_*	3,160	19,222	N/A
α	1.501*±*0.023	1.171*±*0.004	N/A
Time int	04/12/27 –05/06/24	04/12/27–05/06/30	04/12/27–05/06/17
*A_tot_*	126,879,803	2,649,097	225,022,112
*N_tot_*	1,556,626	28,965	768,882
α	1.515*±*0.002	1.278*±*0.006	1.205*±*0.002
μ°	8.055*±*0.078	36.367*±*0.900	9.972*±*0.148
τ°	1.985*±*0.069	27.770*±*0.956	3.271*±*0.135
μ*	7.389*±*0.142	10.250*±*1.440	9.533*±*0.190
τ*	1.687*±*0.079	9.480*±*0.666	2.822*±*0.106

Each data set contains the amount of each individual donation together with the date of donation. gives the total amount of donations (local currency) in the given time interval (top: before, bottom: after the tsunami), the total number of donations, respectively. *α* is the exponent of the power law, Eq. (1) together with the standard error *σ*. *μ* and *τ* are the fit parameters of Eqs. (2), (3) together with their standard errors. The two different values of *μ* and *τ* are obtained from the two different time series for the the number (*°*) and the amount (*) of donations.

**Figure 1:**
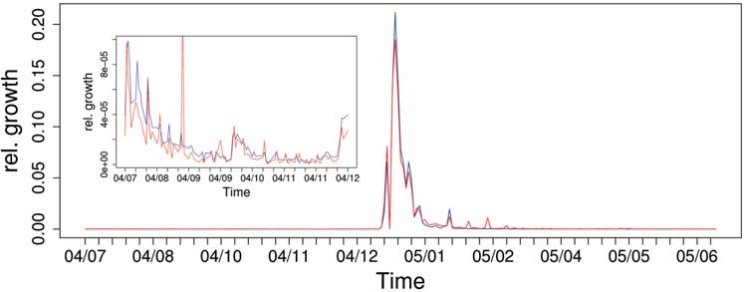
Daily number (blue) and amount (red) of donations shown as a fraction of the total number/amount over a period of one year (mid of 2004 until mid of 2005, time series DH, see Table 1). The inset magnifies the relative growth of number and amount of donations for the half-year period preceeding the earthquake.

For the largest of these time series (DH), we also compared the number and amount of donations for an interval of six months *before* and *after* the tsunami (see Fig. 1). The vast relative growth for both amount/number of donations occured within a period of 3 weeks after the catastrophe (2005/12/26), small peaks in late January 2005 are due to aggregated donations that have been collected from larger groups before transferring them to the donor organization. Because the relative growth of the amount and the number of donations coincide most of the time, we later model the dynamics of donations in terms of frequencies only.

**Figure 2:**
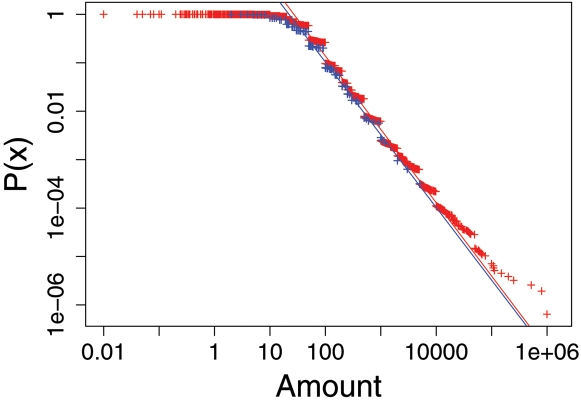
Probability distribution, *P*(*x*), estimated from the relative frequencies to find donations of an amount of *x* or larger, for both time intervals before (blue) and after (red) the tsunami (time series DH, see Table 1).

From the inset of Fig. 1 we further note that, even if the donations after the disaster outperform those before by a number of magnitudes, there are still statistical signs in the data before the disaster. These can be read to compare the distribution of donations before and after the tsunami. Fig. 2 depicts the probability distribution, *P*(*x*), estimated from the relative frequencies to find donations of an amount of *x* or larger, for both time intervals. The plots show a clear power-law like behavior of
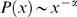
(1)over several orders of magnitude. This indicates the scalefree nature of donations; i.e., there is no typical amount of donations, but the full range of possible values can be found with a definite probability. The plot also indicates that multiples of 10 have a higher preference.

Interestingly, we find that the exponents *α* for the given donor organization (DH) are quite similar both before and after the disaster. This suggests that although the number and amount of donations have changed tremendously, their statistical properties remain almost the same. Consequently, there is an indication for a kind of universal behavior in donating money. The finding can be confirmed also for another German donor organization (AH) which has collected ten times less in terms of number and amount of donations (see Table 1). Comparing this with the Swiss donor organization (GK), we find a a similar, slightly lower value of *α* again (see Table 1).

### Dynamic model of donations

In order to sketch the time dependent evolution of donations after the disaster shown in Fig. 1, we adopt a very simple epidemic model [11], [12] which was also applied to the adoption of new technologies [13] and now proves to be sufficient for describing the observed dynamics in donations. We assume that a fraction *y* of the total population *N* is willing to donate money after the catastrophe, where *y* is treated as an exogeneous parameter that may vary by country (for the tsunami donations, *y* was about 0.1 in Switzerland and about 0.08 in Germany). So gives the total number of *possible* donators in a country. The number of *actual* donators, , is a subset of both *N* and and changes over time. In order to model its dynamics, we assume that a potential donator, *P*, becomes an actual donator, *A*, by interacting with individuals which have already donated. This can be described as a non-local interaction via a mean field that represents the media. In fact, this assumption is quite appropriate as the mass media homogeneously and constantly informed about the disaster and its consequences as well as about the tremendous amount of donations received.

**Figure 3:**
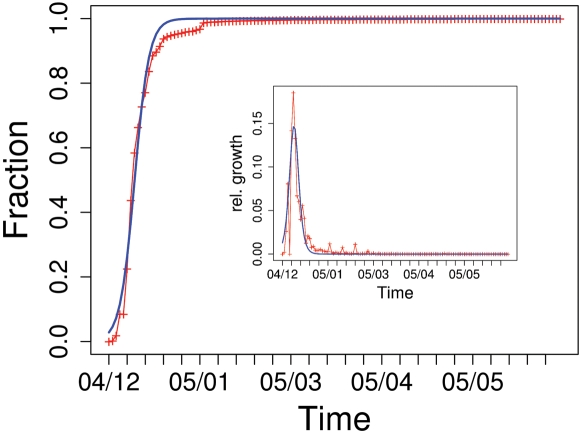
Fraction of the total number of donations (inset: relative growth of amount of donations) over time, after the disaster (time series DH). The blue curves results from fits of Eqs. 3, 4 with *μ* = 8.05*±*0.07, *τ* = 1.98*±*0.06 (inset: *μ* = 7.38*±*0.14, *τ* = 1.68*±*0.07 )

The act of donation is described as a transition of a potential into an actual donator, . This transition may occur at a gross rate that depends on a constant *γ* describing the number of interactions per time interval between *P* and *A* and a factor 0*≤κ≤*1, which is the probability that such an interaction leads to a donation. The rate further depends on the fraction , i.e. the probability of a potential donator to interact with someone who already donated, relative to the size of the population. Eventually, the dynamics for the transition of a potential donator also depends on the “resource” , i.e. those potential donators, who did not donate so far. For the increase of the number of actual donators, it then follows the dynamics
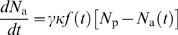
(2)Using the abbreviations for the frequency of actual donators *f*(*t*) and for the time scale , we find eventually the dynamics in the form
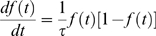
(3)which is known as the logistic equation [19], [20]. Integration leads to the distribution
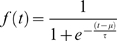
(4)where *μ* gives the time where the relative growth of *f*(*t*) has reached its maximum. Eqs. 3, 4 have been tested against the empirical data obtained from the time series of donations. The data were also used to determine the two “free” parameters *μ*, *τ* of the dynamics (see Table 1). Figure 3 shows, for the largest data set (DH), both the total fraction and the relative growth of donations over time. Despite the very simple dynamics assumed for the model, one realizes a good agreement between theoretical prediction and empirical findings, in particular for the steep rise in the beginning and the saturation phase. The deviations between the estimated fraction and the empirical curve during the end of January 2005 result from the few large donations mentioned as small peaks in the relative growth in Fig. 1.

The data shown in Table 1 indicate that the smallest of the donor organizations (AH) has a much larger time delay in the number of donations which is evidently related to the influence of the mass media. The growth in numbers reached the maximum only after about 36 days, whereas DH, the largest German donor organization, reached this maximum after about 8 days. This is due to the fact that, different from DH, AH was not present in the TV, but mostly supported by their base donators.

**Figure 4:**
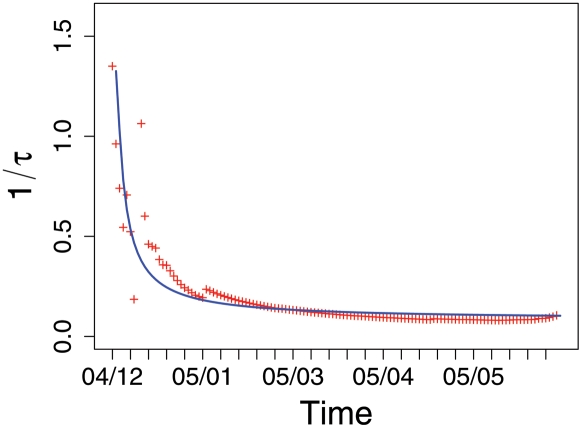
Decay of the parameter 1/*τ* obtained from empirical data of time series DH (red), together with the fit (blue) for 1/*τ* (Eq. 5) resulting in *a* = 0.08*±*0.01, *b* = 2.52*±*0.33, *c* = −1.27*±*0.38.

The simple model of donator dynamics assumes that eventually all possible donators have donated once on an individual basis, i.e. *f*(*t*)→1. It does not consider the subsequent aggregation of donations or a time dependence of the parameter *τ* describing the mean-field interaction between potential and actual donators. We can improve the model further by assuming that the mean-field interaction slows down in the course of time. This can be underpinned by extracting the variation of *τ* from the data in such a way that the theoretical curve matches with the empirical findings. The result shown in Fig. 4 suggests a time dependence of
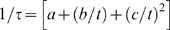
(5)This implies that either or both of the parameters *γ* and *κ* should have decreased their value in the early stage, before they almost reach a saturation level at about 6 weeks after the tsunami. As (*γκ*) gives the number of successful interactions per time interval, this means that in the early stage after the disaster people were more enthusiastic to donate money or could be more easily convinced by the media, while later became more indifferent. So we see the decrease of *τ* in time as an indication of a lack of public interest. Because this interest was mediated by the mass media in a kind of mean-field dissemination, the decrease can be also seen as a decreasing influence of the media when reporting about the aftermath of the disaster and the related donations.

## Discussion

The remarkable findings of our investigations are (i) the statistical similarities in individual donations before and after the tsunami disaster, as well as between two different countries, and (ii) that the collective dynamics of millions of individual donations can be very well described by a simple epidemic model, which has similarities also to the adoption of innovations [13] and saturated growth in biological populations [19]. So, our findings support the idea of certain universality classes for collective dynamics across scientific domains.

The applicability of the SIR dynamics shows that the interaction of the individuals can indeed be modelled by a mean-field interaction which accounts for the dissemination of information by the mass media: the disaster event, broadcasted in the mass media, triggered the first donations, which were then amplified by the mass media again, broadcasting new information both about the disaster and donations received. This resulted in some global feedback dynamics which eventually slowed down both because of a decreasing public interest and a exhausted resource (potential donators). While the latter one sufficiently describes the saturation effect, it was indeed the decreasing public interest and the related influence of the mass media, covered in the model by the time dependent parameter 1/*τ*, which allows to describe the deviations from a simple logistic growth dynamics.
